# On the genome constitution and evolution of intermediate wheatgrass (*Thinopyrum intermedium*: Poaceae, Triticeae)

**DOI:** 10.1186/1471-2148-11-127

**Published:** 2011-05-18

**Authors:** Václav Mahelka, David Kopecký, Ladislava Paštová

**Affiliations:** 1 Institute of Botany, Academy of Sciences of the Czech Republic, Zámek 1, CZ-25243, Průhonice, Czech Republic; 2 Centre of the Region Haná for Biotechnological and Agricultural Research, Institute of Experimental Botany, Sokolovská 6, CZ-77200, Olomouc, Czech Republic

## Abstract

**Background:**

The wheat tribe Triticeae (Poaceae) is a diverse group of grasses representing a textbook example of reticulate evolution. Apart from globally important grain crops, there are also wild grasses which are of great practical value. Allohexaploid intermediate wheatgrass, *Thinopyrum intermedium *(2n = 6x = 42), possesses many desirable agronomic traits that make it an invaluable source of genetic material useful in wheat improvement. Although the identification of its genomic components has been the object of considerable investigation, the complete genomic constitution and its potential variability are still being unravelled. To identify the genomic constitution of this allohexaploid, four accessions of intermediate wheatgrass from its native area were analysed by sequencing of chloroplast *trn*L-F and partial nuclear GBSSI, and genomic *in situ *hybridization.

**Results:**

The results confirmed the allopolyploid origin of *Thinopyrum intermedium *and revealed new aspects in its genomic composition. Genomic heterogeneity suggests a more complex origin of the species than would be expected if it originated through allohexaploidy alone. While *Pseudoroegneria *is the most probable maternal parent of the accessions analysed, nuclear GBSSI sequences suggested the contribution of distinct lineages corresponding to the following present-day genera: *Pseudoroegneria*, *Dasypyrum*, *Taeniatherum*, *Aegilops *and *Thinopyrum*. Two subgenomes of the hexaploid have most probably been contributed by *Pseudoroegneria *and *Dasypyrum*, but the identity of the third subgenome remains unresolved satisfactorily. Possibly it is of hybridogenous origin, with contributions from *Thinopyrum *and *Aegilops*. Surprising diversity of GBSSI copies corresponding to a *Dasypyrum*-like progenitor indicates either multiple contributions from different sources close to *Dasypyrum *and maintenance of divergent copies or the presence of divergent paralogs, or a combination of both. *Taeniatherum*-like GBSSI copies are most probably pseudogenic, and the mode of their acquisition by *Th. intermedium *remains unclear.

**Conclusions:**

Hybridization has played a key role in the evolution of the Triticeae. Transfer of genetic material via extensive interspecific hybridization and/or introgression could have enriched the species' gene pools significantly. We have shown that the genomic heterogeneity of intermediate wheatgrass is higher than has been previously assumed, which is of particular concern to wheat breeders, who frequently use it as a source of desirable traits in wheat improvement.

## Background

A significant proportion of grasses from the wheat tribe Triticeae (Poaceae) is closely linked with the history of human civilization. Apart from the globally important major grain crops wheat, barley and rye, many wild grasses were either grown as primitive crops in the past or have been cultivated for pastoral purposes or rangeland protection to this day. Some even represent an invaluable source of genetic material potentially useful in crop improvement. Intermediate wheatgrass, *Thinopyrum intermedium *(Host) Barkworth et D. R. Dewey, is a predominantly hexaploid (2n = 6x = 42) grass of great practical value. Thanks to its high production, drought and frost tolerance and non-invasiveness, it is excellent as forage and for erosion control in areas with harsh environmental conditions [1]. It also possesses resistance to a number of pests and diseases of wheat. An artificial hybrid between intermediate wheatgrass and wheat, ×*Trititrigia cziczinii *Tsvel., was described by Tsitsin [2] and taxonomically validated by Tsvelev [3]. Because of its crossability with wheat, intermediate wheatgrass has been used extensively as an alien genetic resource for wheat improvement. Many of its desirable traits have been introduced into the wheat genome [4-8].

The economic importance of this hexaploid prompted considerable efforts to identify its genomic components. Despite this, its entire genomic constitution and its potential variability remain unresolved. Earlier studies based on the degree of chromosome pairing at meiosis in artificial hybrids have put forward multiple theories concerning the species' genomic constitution. *Triticum *L. genomes were often thought to be involved in the genome of intermediate wheatgrass [9-11]. However, often controversial conclusions were drawn because of the inability to distinguish between auto- and allosyndetic pairing at meiosis. After researchers recognized the possible role of autosyndetic pairing, more convincing conclusions have been reached. *Thinopyrum intermedium *has been described as a segmental autoallohexaploid, consisting of two closely related, partially homologous, genomes and one distinctly diverse genome, with at least one genome being homologous with *Agropyron elongatum *(Host) P. Beauv. (= *Thinopyrum elongatum *(Host) D. R. Dewey) [12-15]. Löve [16] placed intermediate wheatgrass in the genus *Elytrigia *Desv. According to his treatment, *Elytrigia *polyploids consist of three different basic genomes **J**, **E**, **S**, representing closely related *Thinopyrum *Á. Löve and *Lophopyrum *Á. Löve, and *Pseudoroegneria *(Nevski) Á. Löve haplomes, respectively. The contribution of *Pseudoroegneria *was later confirmed by Liu and Wang [17] and Assadi and Runemark [18]. In the 1990s, the genomic *in situ *hybridization technique (GISH) established itself as a valuable tool for genome structure analyses, making it possible to indicate potential progenitors of polyploid species. Using GISH, Chen et al. [19] examined the genomic constitution of *Th. intermedium*. Their results indicated that it contained three distinguishable chromosome sets designated **J**, **J**^**S **^and **S**, with 17-21, 6-11 and 13-14 chromosomes, respectively. The **J **genome was related to both *Th. elongatum *and *Th. bessarabicum *(Savul. & Rayss) Á. Löve, the **J**^**S **^genome referred to a modified *Th. elongatum*/*Th. bessarabicum *genome, and the **S **genome originated from *Pseudoroegneria strigosa *(M. Bieb.) Á. Löve. Similar conclusions were drawn by Tang et al. [6], who described the genomic composition of *Th. intermedium *as 21**J **+ 7**J**^**S **^+14**S**. Kishii et al. [20] revealed that **V **genome of *Dasypyrum villosum *(L.) P. Candargy (hereafter, genome symbols are according to Wang et al. [21]) could be also involved in the genome of *Th. intermedium *based on GISH. They concluded that a more complex genomic structure is likely in this allopolyploid species, with some potential progenitors still unidentified. Remarkably, a large amount of polymorphism and structure modifications, indicating intrapopulational polymorphism with not all accessions having an identical genomic structure, was observed using GISH [6,19,20] and C-banding [22-24] techniques.

Sequence-based markers represent another potent approach towards disentangling the evolutionary relationships within diverse polyploid complexes, single- (or low-) copy nuclear genes being among the most widely used [25-29]. Granule-bound starch synthase I (GBSSI) was proved to be a single-copy gene in all grasses studied so far [30] and has been successfully employed to examine the origin of several polyploid species [25,26,28,29,31]. On the one hand, GBSSI turned out to be sensitive enough to indicate past introgression [26]. On the other hand, apart from limitations involving duplication and deletion events [32-34], one disadvantage of applying sequence-based markers alone stems from the inability to distinguish whether different gene copies represent true homoeologs representing whole chromosome sets or mere chromosome segments acquired through hybridization or introgression. Sequence-based markers together with *in situ *hybridization are a powerful set of tools for clarifying such complex situations [35]. Along with biparentally inherited nuclear genes, chloroplast markers have been used to identify maternal parents of polyploid species [29,36-39]. Notably, a highly asymmetric pattern of cytoplasmic gene flow has been documented within the Triticeae. *Pseudoroegneria *(**St**) turned out to be the maternal parent in allopolyploids containing the **St **nuclear genome in combination with other genomes [25,36,38,40-42]. Recently, Zhang et al. [43] also provided evidence for cpDNA inheritance from other parents than those containing a **St **nuclear genome.

Despite the high effectiveness of using sequence-based markers in biosystematic studies, they have never been employed to investigate the genomic composition of allohexaploid *Thinopyrum intermedium*. In the present study, we therefore analyse four accessions of hexaploid *Th. intermedium *from its native area in Central Europe (Czech Republic) using (1) chloroplast *trn*L-F sequences to identify which maternal lineage has contributed to the formation of the species; (2) partial GBSSI sequences to identify lineages involved in the formation of its nuclear genome; and (3) genomic *in situ *hybridization to assess the contribution of the putative diploid donor species revealed by *trn*L-F and GBSSI sequences.

## Methods

### Plant material

Four accessions of hexaploid *Thinopyrum intermedium *(Host) Barkworth et D. R. Dewey [syn. *Elytrigia intermedia *(Host) Nevski, *Agropyron intermedium *(Host) P. Beauv.] were analysed. Their choice was based on morphological, flow cytometric, cpDNA and ITS diagnostic markers [44,45] applied in concert to avoid possible inclusion of recent hybrids into the analyses. All samples originated from different parts of the Czech Republic with the aim to cover potential geographic variability: *Thinopyrum intermedium-1*: 3 km NE of Podbořany town, top of Rubín hill, steppe, 50°15.220' N, 13°26.207' E; *Thinopyrum intermedium-2*: Brno town, Kamenný hill, roadside, 49°11.042' N, 16°33.085' E; *Thinopyrum intermedium-3*: 4.5 km N of town Mikulov, steppe, 49°50.425' N, 16°38.417' E; *Thinopyrum intermedium-4*: 4.0 km E of Radějov village, Čertoryje reserve, White Carpathians, mesophilous meadow, 48°51.342' N, 17°24.748' E. Localities of accessions *Thinopyrum intermedium-1*-*3 *correspond to localities 01, 05 and 35 of Mahelka et al. [44]. All accessions are cultivated in the experimental garden at the Institute of Botany of the Academy of Sciences of the Czech Republic in Průhonice, Czech Republic, and herbarium specimens are deposited at the institute's herbarium (PRA).

### Methods

#### DNA extraction and amplification

Genomic DNA was extracted according to [46], but fresh leaves were crushed in liquid nitrogen.

##### *Trn*L-F amplification

The chloroplast *trn*L-F region was amplified for accessions *Thinopyrum intermedium-3 *and *-4 *as described in [45]. Sequences of accessions *Thinopyrum intermedium-1 *and *-2 *were adopted from a previous study [45] (GenBank accession numbers DQ912408 and DQ912410 respectively). PCR products were purified using the QIAquick^® ^PCR purification kit (Qiagen, Hilden, Germany) and directly sequenced (GATC Biotech, Konstanz, Germany) using the primers c, f and e [47]. Electropherograms were edited manually and sequences were deposited in GenBank (*Th. intermedium-3*: GU292419, *Th. intermedium-4*: GU292420).

##### *Granule-bound starch synthase I *amplification

PCR amplifications using two sets of primers (F-for/M-bac, F-for/K-bac [30]), and cloning of PCR products were done as described in [35]. Since F-for/M-bac primers preferentially amplified one gene variant in preliminary analyses, F/M products were sequenced directly in forward and reverse direction with no need for cloning. To eliminate the preferentially amplified gene variant and to retrieve a reasonable proportion of diverse gene variants [see [25]], between 26-40 F/K clones per accession were sequenced using the F-for primer, depending on the variation found within each plant.

#### Alignments and choice of sequences

##### *trn*L-F

Four *Th. intermedium *sequences were aligned along with 46 sequences of monogenomic taxa from throughout the tribe Triticeae downloaded from GenBank (Table 1). Multiple sequence alignment was carried out using the program CLUSTAL_X [48], and the primary alignment was refined manually in BioEdit [49]. The final alignment of 1179 nucleotide sites consisted of the *trn*L(Leu) intron (alignment positions 1-657), the *trn*L gene (3'-exon; 658-708) and the *trn*L-F intergenic spacer (709-1179). The alignment is available as additional file (Additional file 1: Alignment of chloroplast *trn*L-F sequences).

**Table 1:** List of diploid taxa used in the analyses

	GBSSI	*trn*L-*trn*F
*Aegilops*		
*bicornis *Jaub. & Spach	AF079265^30^	EU013485^86^
*comosa *Sibth. & Sm.	AF079263^30^	^a^EU013514^86 b^EU013515^86^
*longissima *Schweinf., Muschl. & Eig	AF079266^30^	EU013620^86^
*markgrafii *(Greuter) K. Hammer	AF079262^30^	AF519111^36^
*speltoides *Tausch	AF079267^30^	AF519112^36^
*tauschii *Coss.	AF079268^30^	AF519113^36^
*umbellulata *Zhuk.	AF079269^30^	EU013680^86^
*uniaristata *Vis.	AF079270^30^	AF519114^36^
*searsii *Feldman & M. Kislev ex K. Hammer		EU013655^86^
*Agropyron*		
*cristatum *(L.) Gaert.	AY011002^75^	AF519116^36^
*mongolicum *Keng	AY011003^75^	AF519117^36^
*Australopyrum*		
*pectinatum *ssp. *retrofractum *(J.W. Vickery) Á. Löve	AF079272^30^	AF519118^36^
*velutinum *(Nees) B.K. Simon	AY011004^75^	AF519119^36^
*Dasypyrum villosum *(L.) P. Candargy	^#a^GU292417^#b^GU292418	AF519128^36^
*Eremopyrum distans *(K. Koch) Nevski	AY011006^75^	AF519150^36^
*Henrardia persica *(Boiss.) C.E. Hubb.	AF079276^30^	AF519152^36^
*Heteranthelium piliferum *Hochst. ex Jaub. & Spach	AF079277^30^	AF519153^36^
*Hordeum*		
*bogdanii *Wilensky	AB154358*	AJ969267^37^
*brachyantherum *Nevski		AF519120^36^
*brachyantherum *Nevski ssp. *californicum *(Covas & Stebbins) Bothmer, N. Jacobsen, Seberg	AF079273^30^	
*brevisubulatum *(Trin.) Link	AY010961^75^	AF519121^36^
*brevisubulatum *ssp. *violaceum *(Boiss & Huet) Tzvelev	AY010964^75^	
*bulbosum *L.	AY010962^75^	AF519122^36^
*comosum *J. Presl		FM163617^87^
*euclaston *Steud.		AJ969355^37^
*marinum *Huds.	AY010959^75^	AF519124^36^
*murinum *L.	AY010960^75^	AF519125^36^
*pusillum *Nutt.	EU282321^26^	AF519127^36^
*spontaneum *K. Koch	AY349349^84^	AJ969296^37^
*vulgare *L.	AB087716^85^	AJ969295^37^
*Peridictyon sanctum *(Janka) Seberg et al.	AF079278^30^	AF519154^36^
*Psathyrostachys*		
*fragilis *(Boiss.) Nevski	AF079279^30^	AF519169^36^
*juncea *(Fisch.) Nevski	AF079280^30^	AF519170^36^
*Pseudoroegneria*		
*libanotica *(Hack.) D.R. Dewey	AY360824^25^	AF519156^36^
*spicata *(Pursh) Á. Löve	^a^AY010991^75 b^AY011000^75^	AF519158^36^
*spicata *ssp. *inermis *(Scribn., and J.G. Smith) Á. Löve		AF519157^36^
*strigosa *(M. Bieb.) Á. Löve	AY360823^25^	
*strigosa *subsp. *aegilopoides *(Drobov) Á. Löve		AF519155^36^
*tauri *(Boiss. & Balansa) Á. Löve	EU282326^26^	EF396991*
*Secale*		
*cereale *L.	AY011009^75^	AF519162^36^
*montanum *Guss.	AF079282^30^	AF519161^36^
*strictum *C. Presl ssp. *anatolicum *(Boiss.) K. Hammer	AY011008^75^	AF519163^36^
*Taeniatherum caput-medusae *(L.) Nevski	^a^AY011010^75 b^AY360848^25^	AF519164^36^
*Thinopyrum*		
*bessarabicum *(Savul. & Rayss) Á. Löve	AF079283^30^	AF519165^36^
*elongatum *(Host) D. R. Dewey	AF079284^30^	AF519166^36^
*Triticum*		
*boeoticum *Boiss.	AF079285^30^	AF519168^36^
*monococcum *L.	AF079286^30^	EU013665^86^
*urartu *Thumanjan ex Gandilyan	AF079287^30^	EU013674^86^
*Bromus tectorum *L.	AY362757^25^	
*Bromus sterilis *L.	EF656589^29^	

List of taxa used in the analyses with their GenBank accession numbers. Superscript letters after accession numbers refer to the source articles, * - unpublished. ^a ^and ^b ^refer to different accessions of the same species, ^#a, #^^b ^- sequences from the same individual amplified in this study. For outgroups, see Methods.

##### GBSSI

Amplified GBSSI sequences of each plant were aligned separately with Clustal_X and corrected in BioEdit. Since *Th. intermedium *is allohexaploid, several divergent homoeologous sequence types were amplified in each plant. The main objective of this study was to identify the origin of diverse homoeologous copies in the allopolyploid rather than analysing the variation found within each accession in detail. Therefore, unique substitutions (singletons, i.e., phylogenetically uninformative polymorphic sites in which a rare base is found in only one of the sequences) were omitted when assigning sequences to groups. Sequences displaying a mosaic sequence pattern, i.e., combining different parts typical of individual sequence groups, were considered recombinant and excluded from the analyses. Only one sequence per group displaying the least number of singletons was included in the analyses. A list of 18 *Th. intermedium *GBSSI sequences used in phylogenetic analyses including their GenBank accession numbers is presented in Table 2.

**Table 2:** Clones representing different GBSSI variants as inferred from phylogenetic analyses

Sequence	GenBank	Inferred origin
*Plant 1*		

*Th. intermedium*-1a*	GU292399	*Taeniatherum*
F/K clones (40/8)		
*Th. intermedium*-1b (10)	GU292400	*Dasypyrum*
*Th. intermedium*-1c (19)	GU292401	*Thinopyrum*
*Th. intermedium*-1d (3)	GU292402	*Pseudoroegneria*

*Plant 2*		

*Th. intermedium*-2a*	GU292403	*Taeniatherum*
F/K clones (26/3)		
*Th. intermedium*-2b (17)	GU292404	*Dasypyrum*
*Th. intermedium*-2c (5)	GU292405	*Dasypyrum*
*Th. intermedium*-2d (1)	GU292406	*Dasypyrum*

*Plant 3*		

*Th. intermedium*-3a*	GU292407	*Taeniatherum*
F/K clones (34/10)		
*Th. intermedium*-3b (9)	GU292408	*Dasypyrum*
*Th. intermedium*-3c (1)	GU292409	*Dasypyrum*
*Th. intermedium*-3d (2)	GU292410	*Aegilops*
*Th. intermedium*-3e (11)	GU292411	*Thinopyrum*
*Th. intermedium*-3f (1)	GU292412	*Pseudoroegneria*

*Plant 4*		

*Th. intermedium*-4a*	GU292413	*Taeniatherum*
F/K clones (32/4)		
*Th. intermedium*-4b (20)	GU292414	*Dasypyrum*
*Th. intermedium*-4c (2)	GU292415	*Pseudoroegneria*
*Th. intermedium*-4d (6)	GU292416	*Aegilops*

Sequences representing different GBSSI variants amplified in four *Thinopyrum intermedium *accessions using the F/M and F/K primers. Sequences marked with an asterisk were amplified with F/M primers and directly sequenced. The numbers of sequenced F/K clones and putative recombinants are provided for each accession. Sequence identifier, GenBank accession number and inferred sequence origin are given. After each sequence identifier, the number of identical clones amplified in each accession is given in parentheses.

Representative accessions of monogenomic diploid taxa from throughout the tribe Triticeae plus two *Bromus *L. accessions used as an outgroup were retrieved from GenBank (Table 1) and aligned with the *Th. intermedium *sequences using Clustal_X. The final alignment was improved manually in BioEdit. Intriguingly, two very different GBSSI sequences of *Dasypyrum villosum *were downloaded from GenBank (AF079274 and AY556480). These sequences were excluded from the dataset because they appeared in different parts of the phylogenetic tree in preliminary analyses. We replaced them with two newly amplified F-for/K-bac sequences from one individual of *D. villosum *(USDA accession identifier PI639751). All procedures including DNA extraction, PCR amplification, cloning and sequencing were done as described for *Th. intermedium*. Out of ten sequences, two slightly different clones (GU292417 and GU292418), matching the sequence AF079274 in exploratory phylogenetic analysis, were found and used in phylogenetic analyses.

Alignment of *Th. intermedium *sequences and other Triticeae was straightforward in all exon regions and a major part of intron 9, but ambiguous in two regions. Firstly, in intron 10, two strongly divergent sequence types were present. Since positional homology across all sequences could not be assigned in this intron, sequences of each type were aligned separately, resulting in two separate indels of 100 and 75 bp. Secondly, an insertion/deletion (indel) region consisting of repetitive motifs in intron 9 at alignment positions 93-109 was ambiguous as well and was therefore excluded from the analyses. Since all but four sequences were amplified with the F-for/K-bac primers, the four F-for/M-bac sequences were cut so that the final dataset of 65 sequences had 718 nucleotide sites and consisted of partial exon 9 (alignment sites 1-81), intron 9 (82-189), complete exon 10 (190-369), intron 10 (370-582), and partial exon 11 (583-718). The alignment is available as additional file (Additional file 2: Alignment of nuclear GBSSI sequences). Exon/intron boundaries were determined by comparison with the representative sequences from the Poaceae [30].

#### Phylogenetic analyses

##### General approach

To place the *trn*L-F and GBSSI sequences obtained from *Th. intermedium *in a phylogenetic context within the Triticeae, two phylogenetic reconstruction methods were employed for each marker: Bayesian analysis and maximum parsimony (MP) analysis. Prior to the phylogenetic analyses, the potential phylogenetic information contained in indel regions was examined in several preliminary analyses. Unambiguous indels were coded following the Modified complex indel coding (MCIC) method of Müller [50], whereby the phylogenetic information contained in indels was implemented into data matrices. For the purpose of MP analyses, this was done automatically using the program SeqState [51], generating a NEXUS output file, which can be readily executed in PAUP* 4b10 [52]. For Bayesian analyses, the extension of the nucleotide matrix of the NEXUS output file containing coded indels as characters was manually added to the nucleotide data matrix. The data file was then analysed as a combined dataset, consisting of DNA (nucleotide characters) and standard (indel characters) data. Coding of indels did not have a great effect on the resulting tree topologies, but it increased topological robustness within some clades in which *Th. intermedium *sequences appeared. Coding of indels was applied in all analyses except for the Bayesian analysis of *trn*L-F sequences, in which it did not improve results.

##### GBSSI

Bayesian phylogenetic analysis was undertaken using MrBayes 3.1.2 [53,54] as follows: (i) The model of molecular evolution that best fit the DNA data partition was determined with MrModeltest 2.3 [55]; (ii) According to the SYM + G model determined by the hierarchical Likelihood Ratio Tests (hLRTs), six substitution rates and gamma distribution were specified as settings; (iii) *Bromus tectorum *L. was used as an outgroup; (iv) Two simultaneous Metropolis coupled MCMC analyses with four chains each were run, incrementally heated by a temperature of 0.1 for 3.5 million generations, and every 100th tree was sampled; (v) After stationarity was reached, the first 25% trees were discarded as burn-in, and a consensus tree with branch lengths and posterior probabilities was computed. The MP analysis was run in PAUP* as heuristic searches with 10 random addition replicates, tree bisection-reconnection (TBR) branch swapping, and keeping no more than 100 trees of length greater than or equal to 1 in each replicate. *Bromus tectorum *and *B. sterilis *L. were used as an outgroup. A 85% majority-rule consensus tree was constructed. As a measure of topological robustness, bootstrapping was carried out with 1000 replicates using the same settings.

##### *Trn*L-F

Phylogenetic analyses were performed as described for GBSSI, with the following modifications: Bayesian analysis - (i) According to the F81 + I + G model, one substitution rate and gamma distribution with a proportion of invariable sites were specified as settings; (ii) *Psathyrostachys fragilis *(Boiss.) Nevski was used as the outgroup; (iii) The analysis was run for 3 million generations. MP - (i) *Psathyrostachys fragilis *and *P. juncea *(Fisch.) Nevski were used as the outgroup.

#### Sequence divergence and estimation of functional role of *Th. intermedium *GBSSI sequences

Coding sequences were translated using BioEdit and checked for stop codons. Sequences which contained stop codons were excluded from further analyses. Pairwise distances between representative GBSSI sequences were calculated using MEGA4 [56] with Kimura 2-parameter (K2P) method and tabulated. Positions containing alignment gaps and missing data were eliminated only in pairwise sequence comparisons (Pairwise deletion option). We used ratios of non-synonymous substitutions per non-sysnonymous sites to synonymous substitutions per synonymous sites (Ka/Ks) in protein-coding portions of the sequences as an indicator of adaptive molecular evolution [57]. The Ka/Ks ratios, as described by Liberles [58] were calculated using an online server [59]. An excess of non-synonymous substitutions (Ka/Ks > 1) is an indicator of positive selection, while an excess of synonymous substitutions (Ka/Ks < 1) indicates purifying selection imposed by functional constraints. Neutral evolution is reflected in ratios near 1. To determine the evolutionary rates of the representative F/K sequences, substitution rate heterogeneity among coding sequences was examined using Tajima's relative rate test [60] by using MEGA4 with *Bromus tectorum *sequence (AY362757) as the outgroup. The test compares two sequences with an outgroup sequence by counting unique substitutions in both sequences. The molecular clock hypothesis can be rejected if one of the sequences accumulates a significantly larger number of substitutions.

#### Genomic *in situ *hybridization

Genomic *in situ *hybridization (GISH) was used to analyse the contribution of presumed progenitors of four accessions of *Th. intermedium*. At least five metaphase spreads for each of the four accessions were analysed. Using the Biotin-Nick Translation Kit or the DIG-Nick Translation Kit (Roche, Indianapolis, IN) we labeled total genomic DNA of the following species: *Pseudoroegneria spicata *(Pursh) Á. Löve (USDA accession identifier PI563869), *Dasypyrum villosum *(L.) P. Candargy (PI639751), *Taeniatherum caput-medusae *(L.) Nevski (PI598389), *Thinopyrum elongatum *(PI531718), and *Aegilops tauschii *Coss (PI542278). The selection of species used as probes was based on the GBSSI-based phylogeny (see results). All these species were confirmed to be diploids by chromosome counts (data not shown). Seeds of the accessions were kindly provided by the Germplasm Resources Information Network (GRIN) of the United States Department of Agriculture (USDA). *In situ *hybridization and detection were done as described in [35] under conditions of 77% stringency. Slides were evaluated under an Olympus AX70 microscope equipped with epi-fluorescence and a SensiCam B/W camera. ScionImage and Adobe Photoshop software were used for processing of color images. Reprobing of the slides was applied according to [61].

## Results

### Chloroplast *trn*L-F

**Figure 1:**
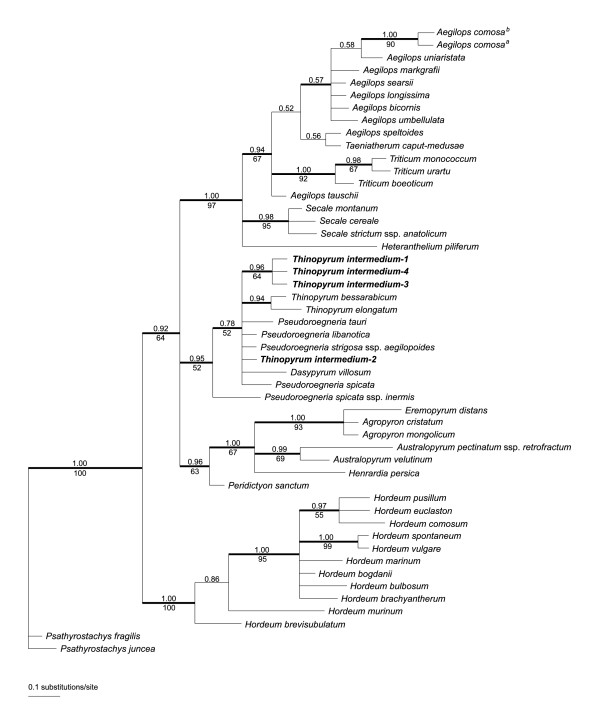
**Bayesian phylogenetic tree based on the chloroplast *trn*L-F region**. *Thinopyrum intermedium *sequences are in bold. Branches found in both Bayesian and maximum parsimony (MP) 85% majority-rule consensus trees are indicated in bold lines. Numbers above and below branches are Bayesian posterior probabilities and bootstrap values for MP, respectively. For GenBank accession numbers, see Methods and Table 1.

Sequences of accessions *Thinopyrum intermedium-1*, *-3 *and *-4 *were identical and differed from the sequence of accession *Th. intermedium-2 *by one substitution. The matrix of 50 sequences comprised 1179 characters, 1098 of which were invariant and 51 were parsimony-informative. When unambiguous indels were converted into coded characters (for MP analysis), the final matrix contained 1207 characters, of which 1098 were invariant and 67 were parsimony-informative. Both phylogenetic analyses resulted in virtually identical topologies of the major Triticeae clades as well as with respect to the phylogenetic relationships of *Th. intermedium *sequences within the Triticeae. MP analysis resulted in 1000 equally most parsimonious trees with a length of 151 steps (CI = 0.815, RI = 0.920). The results of both analyses are summarized in Figure 1. *Thinopyrum intermedium *sequences were placed in a clade comprising species of the genera *Pseudoroegneria*, *Dasypyrum *and *Thinopyrum*, suggesting three possible candidates to be maternal donors. Closer inspection of the alignment revealed that *Th. intermedium *sequences were most similar to *Pseudoroegneria*, and the sequence *Thinopyrum intermedium-2 *was identical with that of *Pseudoroegneria libanotica *(Hack.) D.R. Dewey. Therefore, *Pseudoroegneria *most probably represents the maternal parent of all *Th. intermedium *accessions analysed.

### Nuclear granule-bound starch synthase I

PCR with F-for/M-bac primers yielded fragments of about 1200 bp in all four accessions. Direct sequencing confirmed that this primer combination amplified preferentially one gene variant. In all accessions, intra-individual polymorphism, likely representing allelic variation, was consistently encountered at two sites. By contrast, the F-for/K-bac primer combination amplified heterogenous amplicons of about 650 bp. In total, 132 F/K clones were sequenced, out of which 25 were identified as recombinant and excluded (Table 2), and 18 representative sequences were used for phylogenetic analyses (see Methods and Table 2). Four divergent sequence types were detected in accessions *Thinopyrum intermedium-1*, *-2 *and *-4*, and six in accession *Thinopyrum intermedium-3 *(Table 2). The sequences *Thinopyrum intermedium-2d*, *-3c *and *-3f *were unique within the datasets of individual accessions, and the remaining sequence types were encountered at least twice in each accession (Table 2).

**Figure 2:**
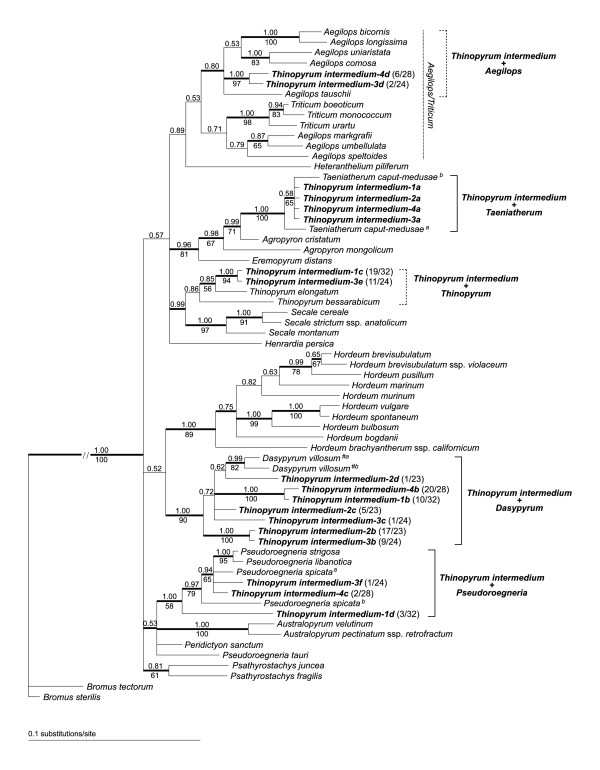
**Bayesian phylogenetic tree based on the GBSSI sequences**. Branches found in both Bayesian and maximum parsimony (MP) 85% majority-rule consensus trees are indicated by bold lines. *Thinopyrum intermedium *sequences are in bold. Clone designations refer to individual plants analysed (numerical identifiers) and individual clones of each plant (letters). After each clone identifier, the number of identical clones and the total number of clones sequenced for that accession is given in parentheses, see also Table 2. The numbers above and below branches are Bayesian posterior probabilities and bootstrap values for MP, respectively.

When indels were converted into coded characters, the final matrix contained 743 characters with 453 invariant and 167 parsimony-informative sites. Both phylogenetic analyses produced congruent trees as to the placement of *Th. intermedium *sequences within the Triticeae. The MP analysis resulted in 800 most parsimonious trees with a length of 641 steps (CI = 0.618, RI = 0.747). The results are summarized in Figure 2.

Direct F/M sequences of all four accessions formed a clade together with diploid *Taeniatherum caput-medusae *(L.) Nevski (Figure 2). F/K sequences grouped with the following diploids: *Aegilops *L., *Thinopyrum*, *Dasypyrum *and *Pseudoroegneria*, not all accessions being represented in every clade. Only the *Dasypyrum *clade consistently comprised F/K sequences of all four accessions. The *Dasypyrum *clade also comprised the highest diversity of *Th. intermedium *sequences, with three different sequence types: sequences *Thinopyrum intermedium-1b *and *4b *were clearly distinguishable from sequences *2c*, *2d *and *3c*, and sequences *2b *and *3b *formed another group. The *Pseudoroegneria *clade comprised sequences of only three accessions: *Thinopyrum intermedium-1*, *-3 *and *-4*. Whereas sequences *3f *and *4c *were most similar to *P. spicata*^a^, sequence *1d *was sister to the remainder of the *Pseudoroegneria *clade and likely represented a different gene variant. Apart from the *Taeniatherum*, *Dasypyrum *and *Pseudoroegneria *clades, GBSSI sequences of *Th. intermedium *fell into two additional, moderately supported clades. Sequences *3d *and *4d *grouped with *Aegilops bicornis *Jaub. & Spach, *A. longissima *Schweinf., Muschl. & Eig, *A. uniaristata *Vis., *A. comosa *Sibth. & Sm. and *A. tauschii *Coss. and formed a subclade of the whole *Aegilops/Triticum *alliance. The last clade in which *Th. intermedium *sequences appeared was formed by *Thinopyrum elongatum *and *Th. bessarabicum*, and *Th. intermedium-1c *and *3e*. Though the clade has only moderate support, *Th. elongatum*/*bessarabicum *sequences are clearly the most similar ones.

According to the inferred origins of the *Th. intermedium *F/K sequences (Table 2, Figure 2), the most frequently amplified sequence type was that of *Dasypyrum *with 63 sequences out of 107, followed by *Thinopyrum *(30/107), *Aegilops *(8/107) and *Pseudoroegneria *(6/107). Interestingly, all F/K sequences of accession *Thinopyrum intermedium-2 *fell into the *Dasypyrum *clade.

### Sequence divergence and estimation of functional role of *Th. intermedium *GBSSI sequences

Protein translations revealed stop codons in exon 13 in all four F/M sequences, indicating that the *Taeniatherum*-like gene variants are probably non-functional. Moreover, all F/M sequences contained a 10-bp deletion in exon 11, where the reverse K-bac primer was designed [see [25]]. These pseudogenic sequences were excluded from further analyses. Pairwise distances between the representative F/K clones are tabulated (Table 3). Two pairs of identical sequences were encountered (*Thinopyrum intermedium-1c*/*3e *and *2b*/*3b*). Distances between the remaining sequences ranged from 0.003 (sequences *1b*/*4b*) to 0.068 (*3f*/*4b*). When *Th. intermedium *sequences were analysed for Ka/Ks ratios, a positive selection along branches leading to *Thinopyrum intermedium-4b, 3c *and *4c *sequences was detected. Additionally, a positive selection along the branch leading to the *Thinopyrum intermedium-3c*/*2d *ancestor was detected (see Additional file 3: Summary statistics for Ka/Ks analysis). In all other cases the Ka/Ks ratio was < 1, suggesting that the purifying selection prevailed among the sequences tested. Tajima's relative rate test could not be calculated between the two above-mentioned pairs of substitutions-free sequences. The test revealed significant rate heterogeneity at the 5% level between the sequences *Thinopyrum intermedium-3f *and *4c *(data not shown). All other comparisons (88) exhibited non-significant rate heterogeneity, indicating approximate rate equivalence among the lineages.

**Table 3:** Pairwise distances among GBSSI sequences.

	1b	1c	1d	2b	2c	2d	3b	3c	3d	3e	3f	4b	4c	4d
1b														
1c	0.059													
1d	0.063	0.035												
2b	0.044	0.048	0.053											
2c	0.029	0.044	0.053	0.027										
2d	0.031	0.054	0.060	0.035	0.027									
3b	0.044	0.048	0.053	0.000	0.027	0.035								
3c	0.037	0.047	0.060	0.033	0.022	0.031	0.033							
3d	0.050	0.034	0.048	0.046	0.037	0.046	0.046	0.037						
3e	0.059	0.000	0.035	0.048	0.044	0.054	0.048	0.047	0.034					
3f	0.065	0.046	0.044	0.049	0.053	0.056	0.049	0.060	0.053	0.046				
4b	0.003	0.064	0.067	0.047	0.032	0.035	0.047	0.040	0.055	0.064	0.068			
4c	0.059	0.033	0.038	0.048	0.047	0.050	0.048	0.054	0.040	0.033	0.012	0.063		
4d	0.053	0.036	0.050	0.048	0.040	0.048	0.048	0.040	0.009	0.036	0.055	0.057	0.042	

Pairwise distances between representative GBSSI sequences amplified in four *Thinopyrum intermedium *accessions, computed using Kimura 2-parameter method (pairwise deletion option).

### Genomic *in situ *hybridization

**Figure 3:**
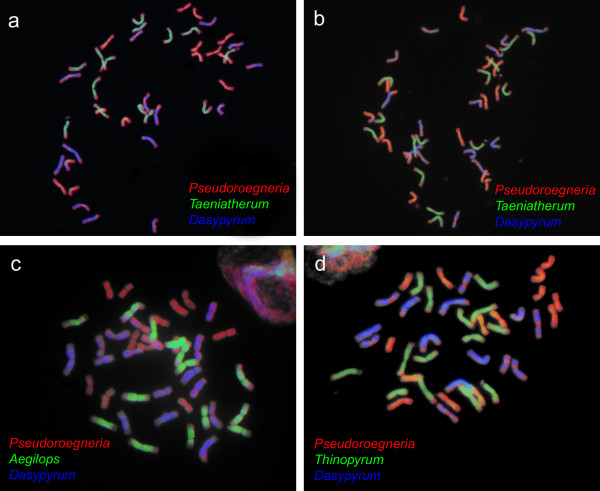
**Molecular cytogenetic analysis of *Thinopyrum intermedium***. Molecular cytogenetic analysis of accessions *Thinopyrum intermedium-2 *(a, c and d) and *Thinopyrum intermedium-3 *(b). (a, b) Fluorescent signals of total DNA of *Pseudoroegneria spicata *labeled with digoxigenin (red pseudocolor), total genomic DNA of *Taeniatherum caput-medusae *labeled with biotin (green pseudocolor) and total genomic DNA of *Dasypyrum villosum *(blue pseudocolor) labeled with digoxigenin after washing and reprobing of the slide. Each of these three probes produced dispersed signal over 14 chromosomes, presumably representing individual subgenomes. (c, d) Fluorescent signals of total genomic DNA of *P. spicata *labeled with digoxigenin (red pseudocolor) and total genomic DNA of *D. villosum *labeled with biotin (blue pseudocolor), and, after washing and reprobing, total genomic DNA of *Aegilops tauschii *(c) labeled with biotin (green pseudocolor) and total genomic DNA of *Thinopyrum elongatum *(d) labeled with digoxigenin (green pseudocolor). Note the overlapping signal of *T. caput-medusae*, *Th. elongatum*, and *Ae. tauschii *on one subgenome.

GISH with each of the genomic DNA of *Pseudoroegneria spicata*, *Dasypyrum villosum*, *Thinopyrum elongatum*, *Aegilops tauschii *and *Taeniatherum caput-medusae *produced dispersed signal over the 14 chromosomes of *Thinopyrum intermedium *(Figure 3a-d). Both *P. spicata *and *D. villosum *produced signal on separate chromosome sets, presumably representing two distinct subgenomes (**St **and **V**) of *Th. intermedium*. Labeled DNAs from *Thinopyrum elongatum*, *T. caput-medusae *and *A. tauschii *produced overlapping signal on the remaining chromosome set (Figure 3a-d), suggesting that the chromosomes of the third subgenome are closely related to all three diploids. Proper identity of the third subgenome therefore remains unclear. Interestingly, all chromosomes of this subgenome carried terminal translocations from *P. spicata*. Similarly, several chromosomes belonging to the subgenome of *D. villosum *displayed signal from *P. spicata *in pericentromeric and subtelomeric regions. GISH produced identical results in all four accessions analysed.

## Discussion

*Thinopyrum intermedium *is a grass of vast practical utility. In particular, it has been used as a source of desirable traits in wheat breeding programmes [8]. Understanding its genomic composition is therefore of great interest. While numerous studies described the genomic structure of this allohexaploid using cytogenetic methods, the present study provides a new insight into the genome structure based on sequencing followed by genomic *in situ *hybridization (GISH). It is hypothesized that the donor of chloroplast DNA is the maternal parent of *Th. intermedium *and that the different GBSSI variants represent homoeologous copies contributed to *Th. intermedium *by its putative progenitors. GISH was used to confirm the contribution of the putative progenitors revealed by sequence data.

### Maternal origin

Chloroplast *trn*L-F sequences indicate *Pseudoroegneria *as the likeliest maternal progenitor of the four accessions of *Th. intermedium *analysed. The presence of *Pseudoroegneria*-derived chloroplast sequences is consistent with the GBSSI data, according to which *Pseudoroegneria *is one of the progenitors of *Th. intermedium-1*, *-3*, and -*4*. GISH further confirmed the contribution from *Pseudoroegneria *to all accessions studied.

An asymmetric pattern of cytoplasmic gene flow has been documented in other Triticeae allopolyploids. *Pseudoroegneria *(**St**) was the maternal parent of polyploids containing the **St **nuclear genome in combination with other genomes [[40] and references therein]. This phenomenon was further documented in numerous cases, e.g., in North American and Eurasiatic *Elymus *species [25,36,38,41,42]. Recently, Zhang et al. [43] examined the maternal origins of fourteen *Kengyilia *(**StYP**) species and found that both *Pseudoroegneria *and *Agropyron *(**P**) are the likely maternal genome donors to the species under study, providing evidence for cpDNA inheritance from another parent than the one containing the **St **nuclear genome. If *Pseudoroegneria *really is the maternal parent of *Th. intermedium*, then it is consistent with most Triticeae allopolyploids in which *Pseudoroegneria *was the maternal parent and also contributed to the nuclear genome. The identity of chloroplast DNA in *Th. intermedium *should be verified by additional chloroplast regions.

### Sequence divergence and estimation of functional role of *Th. intermedium *GBSSI sequences

The primary goal of the closer inspection into the evolution of the sequences was to determine whether the different GBSSI gene variants are functional (and possibly which). The *Taeniatherum*-like copies amplified with F/M primers were most probably non-functional pseudogenes as they contained stop codons as well as a deletion in exon region, and thus were not analysed further. In F/K clones, we used the ratio of non-synonymous to synonymous substitution rates (Ka/Ks) as an indicator of molecular adaptation [57]. It is important to underline here that we only worked with a portion of the GBSSI gene, which may not necessarily reflect the gene in its entirety. While the analysis clearly showed that purifying selection prevailed among the sequences tested, a positive selection has occurred too. The relative rate test further revealed approximate rate equivalence among all the pairs of lineages but one. It is difficult to speculate whether the relaxation of selective constraints encountered in a portion of GBSSI sequences is indicative of gene duplication, potential neofunctionalization or eventual pseudogenization. Kondrashov et al. [62] showed that both orthologous genes and similarly diverged recent paralogs were the subject of purifying selection; however, purifying selection acting on paralogs was substantially weaker than purifying selection affecting unduplicated orthologs. While a deep analysis of the GBSSI sequences could answer some of these questions, such an analysis is far beyond the scope of this paper and represents an interesting topic in its own right. As constrained sequences are supposed to be functional, we provisionally consider the majority of the F/K sequences as representing functional gene variants.

### Nuclear genome composition

Our GBSSI data indicate the contribution of distinct lineages falling to the following present-day genera: *Pseudoroegneria*, *Dasypyrum*, *Taeniatherum*, *Aegilops *and *Thinopyrum*. The contribution of *Aegilops *and *Thinopyrum *is still uncertain due to only moderate support in phylogenetic analyses. GISH clearly identified the donors of two subgenomes: *Pseudoroegneria *and *Dasypyrum*. However, GISH did not provide a clear picture as to the contribution from *Aegilops*, *Thinopyrum *and *Taeniatherum*. Since the presence of five lineages (or even more if we consider multiple contributions from *Dasypyrum*) is not consistent with hexaploidy in *Th. intermedium*, it seems that the origin of *Th. intermedium *is more complex than would be expected if it originated through allohexaploidy alone. So, to explain the diversity of gene copies amplified in the *Th. intermedium *samples studied here (i.e., the number of potential progenitors as well as the sequence diversity within clades in which *Th. intermedium *sequences appear), mechanisms other than allopolyploidy through recent hybridization and/or introgression must also be considered.

For example, the appearance of polymorphism through ancient hybridization (many early hybridizations must have occurred in the early Triticeae) followed by incomplete sorting of ancestral polymorphism could lead to intra-specific variation in a diploid and, consequently, in a polyploid. Origin of North American tetraploid *Elymus *species is blurred by unexpected diversity of *Pseudoroegneria*-like GBSSI copies, likely caused by either ancient introgression or incomplete sorting of ancestral polymorphism [63]. The general question is how much of potential intra-individual polymorphism in nuclear genes (in diploids in particular) may have been overlooked. Only extensive sampling of Triticeae diploids would tell how common is this phenomenon.

Gene duplication is another mechanism potentially responsible for excessive gene diversity [28]. *Thinopyrum intermedium *is a species possessing a large amount of cytogenetic polymorphism and structural modifications of chromosomes, with not all accessions previously studied having identical genomic structure [20,22-24]. Therefore, duplications of some loci following allohexaploid formation followed by paralog diversification cannot be ruled out. Corresponding orthologs and paralogs would form two clades that would be more or less similar to one another in a phylogenetic analysis. Since gene loss must also be taken into account, it cannot be ruled out that only paralogous sequences of an individual homoeolog (i.e. progenitor) exist within the *Th. intermedium *genome.

Furthermore, intra-individual variation in a marker may be the result of heterozygosity. Allelic variation is usually irrelevant for disentangling origins of allopolyploid species. However, if allelic variation spans species boundaries, i.e., if some alleles of a species are more closely related to alleles of another species than they are to those of the same species [64], such a variation might confuse the identification of the allopolyploid's progenitors.

#### *Thinopyrum intermedium *and *Pseudoroegneria*

The contribution from *Pseudoroegneria *to the accessions studied here is evidenced by chloroplast and GBSSI markers as well as *in situ *hybridization. *Pseudoroegneria*-like GBSSI variants were amplified in three out of four accessions (though the placement of sequence *Thinopyrum intermedium-1d *in the *Pseudoroegneria *clade is questionable due to only moderate support in the MP analysis); between one and three *Pseudoroegneria*-like sequences were retrieved from the three individuals (Table 2). Such a biased proportion of amplified *Pseudoroegneria*-like copies is not consistent with the contribution of a whole *Pseudoroegneria*-derived genome. However, GISH clearly identified the presence of a whole chromosome set corresponding to *Pseudoroegneria *in all accessions studied. Interestingly, *Pseudoroegneria*-like sequence variant was very rare in the three accessions and may therefore also be present in accession *2*, but maybe was not retrieved by the clones. To achieve a good representation of individual gene variants, we performed PCR in triplicates and mixed equimolar amounts of PCR products prior to cloning. Moreover, biased amplification due to fragment length differences can be excluded, as all fragments amplified with the F/K primers are of similar lengths. Thus, the reason for such underrepresentation of *Pseudoroegneria*-like gene variants is yet unclear.

The presence of the *Pseudoroegneria *subgenome in *Th. intermedium *is concordant with the literature [6,17-19]. Liu and Wang [17] and Tang et al. [6] identified in *Th. intermedium *two pairs of long chromosomes and one pair of short chromosomes, ascribing the long sets of chromosomes to *Thinopyrum *and the short set to *Pseudoroegneria *(**St**). Assadi and Runemark [18] also suggested the presence of one genome of *Th. intermedium *homologous to *Pseudoroegneria *(**St**) based on chromosome pairing in interspecific hybrids.

#### *Thinopyrum intermedium *and *Dasypyrum*

Phylogenetic analyses clearly placed *Th. intermedium *sequences in a clade containing *Dasypyrum *(Figure 2), identifying *Dasypyrum *as one of the progenitors. *Dasypyrum*-like sequences were the most frequently retrieved sequence types overall and were amplified in all four individuals (Table 2). Consistently, GISH identified the presence of a *Dasypyrum*-like genome in all accessions studied (Figure 3). Remarkably, if we omit unique sequences *2d *and *3c*, accessions *Thinopyrum intermedium-1 *and *-4 *harbour *Dasypyrum*-derived sequences different from accessions *-2 *and *-3*. The presence of three different *Dasypyrum*-like sequence types in the four accessions coupled with their relatively high divergence is intriguing. For example, sequence *Thinopyrum intermedium*-*1b *differs from sequence *2c *by 16 substitutions and two indels of 8 and 4 bp (K2P distance 0.029) and from sequence *3b *by 24 substitutions and two indels (0.044). For illustration, the difference between *Thinopyrum intermedium-1b *and *Pseudoroegneria*-like sequence *4c *is 32 substitutions and three indels (0.059). Such diversity of *Dasypyrum*-like sequences could have several explanations: 1) contribution from different sources close to *Dasypyrum *and maintenance of the divergent copies, 2) duplication and diversification of *Dasypyrum*-like sequences following the origin of the allopolyploid, giving rise to divergent paralogs, 3) allelic variation, and 4) a combination of 1-3.

It is hard to explain the first scenario, as three different lineages are one more than the number of currently recognized *Dasypyrum *haplomes. However, apart from the acknowledged existence of two allogamous *Dasypyrum *species, *Dasypyrum villosum *(diploid, haplome **V**) and *D. breviaristatum *(Lindb. f.) Frederiksen (diploid and autotetraploid, haplome **V**^**b **^- [65]), the situation within the genus is yet to be untangled. Investigations of the genome relationships within *Dasypyrum *revealed substantial dissimilarity between the **V **and **V**^**b **^genomes [65-68]. Both the **V **and **V**^**b **^genomes are so unrelated that Uslu et al. [69] suggested a weaker relationship between the two *Dasypyrum *species than of *D. villosum *with *Thinopyrum bessarabicum *and *Secale cereale*. Similarly, Yang et al. [68] showed that the RAPD pattern of *D. breviaristatum *was closer to *Thinopyrum intermedium *than to *D. villosum*. Since no sequence of *Th. intermedium *accessions studied by us is tightly related to present-day *D. villosum *in the phylogenetic tree (Figure 2), the possibility that *D. breviaristatum *or an extinct or other unsampled *Dasypyrum *(or their hybrid) are the ancestral species cannot be ruled out. Discovering potential intra-specific diversity within *Dasypyrum *could therefore at least help clarify the situation as to potential multiple contributions from *Dasypyrum*.

Alternatively, some of the *Dasypyrum*-like sequences may represent divergent paralogs. Positive selection along branches leading to two *Dasypyrum*-like sequences (*4b *and *3c*) was detected (Additional file 3). There were several non-synonymous substitutions encountered within the sequences. It is not clear, however, whether the non-synonymous substitutions are related to any functional role. Therefore, if these sequences really represent divergent paralogs, it is not clear, whether they underwent non-functionalization (silencing by degenerative mutations), neofunctionalization (non-synonymous substitutions providing a beneficial function) or subfunctionalization (partitioning of ancestral functions between duplicates) [70].

A contribution from *Dasypyrum *to *Th. intermedium *was recently proposed by Kishii et al. [20], who using multicolour GISH indicated the presence of a whole subgenome derived from *Dasypyrum*. Similarly to our results, Kishii et al. [20] observed **St **centromeric signal on nine *Dasypyrum*-like chromosomes (see Figure 3a,b). Similar "translocations" were observed in another allohexaploid *Elymus repens*, in which one pair of chromosomes of the *Hordeum *subgenome (**H**) carried a centromeric **H**/**St **translocation. Intriguingly, both centromeres belonged to *Pseudoroegneria *[35]. Apparently, chromosomal rearrangements have occurred in both species.

#### *Thinopyrum intermedium *and *Taeniatherum*

The contribution from *Taeniatherum *to intermediate wheatgrass is a new finding since it was never reported before. Interestingly, an obscure contribution from *Taeniatherum *has been detected using GBSSI sequences in introduced North American as well as native Central European accessions of the closely related allohexaploid *Elymus repens *[25,26,35]. It is noteworthy, according to [25], that all the sequences of the *Taeniatherum*/*E. repens *clade (including *Taeniatherum caput-medusae *itself) were most probably non-functional pseudogenes, suggesting that the loss of function predated the origin of *E. repens*. Originally, Mason-Gamer [25] interpreted the presence of the *Taeniatherum*-like GBSSI gene as a result of introgression, but later the same author [26] put forward another explanation for its acquisition when she doubted the contribution of *Taeniatherum per se *and suggested that *Taeniatherum *itself might have acquired its GBSSI from other species. Our data on *E. repens *[35] are consistent with either of these hypotheses, as we did not find any direct evidence for a recent contribution from *Taeniatherum *using GISH. If the GBSSI copy amplified in *T. caput-medusae *is a pseudogene, too, the question is what is the functional GBSSI variant of *Taeniatherum*. There is a possibility that the pseudogenic GBSSI variant preferentially amplifies not only in the hexaploids *Th. intermedium *and *E. repens *but also in diploid *Taeniatherum*. Hence, the functional variant may not have yet been retrieved. We tried to recover GBSSI using F/M primers in *Taeniatherum*, but amplifications failed several times, probably due to alteration of primer sites.

The situation in *Th. intermedium *seems to be paralleled by that of *E. repens*. The fact that the *Taeniatherum*-like GBSSI copies amplified in *Th. intermedium *are identical with those pseudogenes amplified in *E. repens *casts doubts on the possible contribution of a whole subgenome from *Taeniatherum*. Instead, it is more likely that *Th. intermedium *acquired its *Taeniatherum*-like copies from another diploid progenitor, which therefore must have contained additional GBSSI copies. Since both *E. repens *and *Th. intermedium *share a *Pseudoroegneria*-like progenitor, *Pseudoroegneria *is a good candidate in this case. The ease with which *Th. intermedium *crosses with *E. repens *under field conditions in Central Europe (hence the connection of *E. repens *with the present study; [44,45,71]) leads to another hypothesis, not incompatible with the former scenarios, according to which either species might have obtained the *Taeniatherum*-like GBSSI pseudogene from one another through introgression.

#### *Thinopyrum intermedium *and diploid *Thinopyrum*

*Thinopyrum*-like sequences were the most often retrieved sequence types in accessions *Thinopyrum intermedium-1 *and -*3*, and their absence in the other two is surprising and hard to explain. Since *Th. intermedium *is a polymorphic species displaying structural chromosomal rearrangements and modifications, locus loss in accessions *-2 *and *-4 *is one possible explanation of this phenomenon. Genomes **E**^**e **^and **E**^**b **^of *Thinopyrum elongatum *and *Th. bessarabicum*, respectively, are further genomes whose involvement in hexaploid *Th. intermedium *has most often been discussed in the literature [6,12,14,15,17,19]. There has been a debate on the degree of homology between *Th. bessarabicum *and *Th. elongatum *genomes [14,72-74]. Still, no consensus has been reached in this respect, and the treatment of the two genomes continues to vary among authors.

#### *Thinopyrum intermedium *and *Aegilops*

As the clade consisting of *Th. intermedium *sequences plus five *Aegilops *species is supported only moderately (Figure 2), the statement that *Th. intermedium *contains genetic material derived from *Aegilops *must be considered as provisional. Remarkably, neither *Triticum*/*Aegilops *clades in GBSSI-based phylogenies presented elsewhere [e.g., [25,26,75]] form tight, strongly supported groups, which is likely caused by the fact that neither *Triticum*, *Aegilops *nor *Triticum *+ *Aegilops *are monophyletic [27]. Early investigations [9-11] advanced the hypothesis that *Th. intermedium *has at least one genome homologous with one of the *Triticum *genomes. Since *Triticum aestivum *L. is an allohexaploid constituting of one *Triticum *genome and two different genomes derived from *Aegilops *[27], it is possible that it was one of *Aegilops *which represented the homologous genome. As noted before, however, early works, in which chromosome pairing data (at high ploidy levels in particular) were used as exclusive evidence for or a measure of genomic relationships, must be interpreted with a great deal of caution. Up to now, the presence of neither *Triticum *nor *Aegilops *within the genome of *Th. intermedium *has been reported based on any more sophisticated approach.

While the identity of *Pseudoroegneria*- and *Dasypyrum*-derived subgenomes seems to be relatively straightforward based on the combined GBSSI and GISH data, the identity of the third subgenome remains unresolved satisfactorily. GBSSI sequences suggest the contribution from *Thinopyrum *and *Aegilops *to the accessions studied, placing these two among possible donors. Similarly, GISH with *Th. elongatum*, *T. caput-medusae *and *A. tauschii *probes produced overlapping signal on one chromosome set (Figure 3a-d). Possibly, the level of divergence among *Th. elongatum*, *T. caput-medusae *and *A. tauschii *is below the detection threshold of *in situ *hybridization in this case, making unambiguous identification of the subgenome impossible. If we set aside contribution from *Taeniatherum *(discussed above), the most parsimonious explanation of the origin of the third subgenome is its hybridogenous origin. Possibly, the progenitor was an ancient hybrid or introgressant between species close to *Aegilops *and *Thinopyrum*. Such an ancient origin of *Th. intermedium *(or at least of some of its subgenomes) could then also explain why some of the GBSSI copies did not group tightly with its presumed progenitors in phylogenetic analyses. This may indicate that some of the ancestors no longer exist or that the allopolyploidization happened so long ago that the genes within *Th. intermedium *have already diverged.

### Interspecific hybridization of *Th. intermedium *and its implications

*Thinopyrum intermedium *is able to hybridize with wheat, whereby it has been utilized as an alien genetic resource in wheat breeding programmes. In terms of cross-compatibility, the presence of genetic material from within the *Triticum*/*Aegilops *alliance in *Th. intermedium *germplasm is therefore not unlikely. Hence, the possibility that *Th. intermedium *acquired its *Aegilops*-like GBSSI copies through introgression from wheat at the hexaploid level cannot be ruled out. The crossability, expressed as a quantity of F_1 _seeds, reached up to 62.5% of all pollinated florets in crossing experiments, and backcrosses were achieved [76-79]. An important fact stemming from the crosses between wheat and *Th. intermedium *is that their crossability highly depends on particular cultivars or strains of both wheat and *Thinopyrum *parents. In this respect, hybridization under natural conditions seems to be even more likely because the chance of meeting a compatible sexual counterpart is increased on one side by the great genetic variability within wild populations of perennial *Th. intermedium *and by fluctuating environmental conditions on the other. Natural hybridization between wheat and its wild relatives (i.e., *Aegilops*) does take place [80], and *Th. intermedium *represents another potentially interesting case of gene flow between a crop and its wild relative that might considerably influence the assessment of risks associated with genetically modified wheat. Hybridization and potential introgression between *Th. intermedium *and wild relatives could also have significantly enriched the species' gene pool [45,81].

## Conclusions

Alongside other reticulation phenomena, interspecific hybridization has played a key role in the evolution of the Triticeae, resulting in strong ecological, morphological and genetic similarities among many Triticeae taxa [14,16,82], notably with distinct gene lineages occurring within some polyploid as well as diploid species [83]. Genomes are not discrete units but form a continuum from homology to lack of homology. It is therefore sometimes difficult to reliably identify all potential progenitors of polyploid species. Our genome analysis of allohexaploid intermediate wheatgrass (*Thinopyrum intermedium*) using chloroplast *trn*L-F and partial nuclear GBSSI sequences followed by GISH confirmed the allopolyploid origin of the species and revealed new aspects in its genomic composition. The data suggested the contribution of distinct lineages falling into five present-day genera: *Pseudoroegneria*, *Dasypyrum*, *Taeniatherum*, *Aegilops *and *Thinopyrum*. Our results, based on four accessions originating from a small geographic region, showed that the genomic heterogeneity of intermediate wheatgrass exists and is higher than has been previously assumed. *Thinopyrum intermedium *is a perennial, out-pollinating grass that is able to hybridize with several other Triticeae grasses including wheat. Transfer of genetic material via extensive hybridization and introgression of *Th. intermedium *with other grasses could have significantly enriched the species' gene pool. Therefore, potential geographical diversity of the species due to, for example, multiple origin and locally-specific hybridizations, can be expected. In this respect, further research should focus on elucidating the genomic composition of *Th. intermedium *across a larger geographic area in context with its ecological adaptation to diverse habitat types. Resolving the genome structure of intermediate wheatgrass is of concern to wheat breeders in particular, who often use it as a source of desirable traits in wheat breeding programmes.

## Authors' contributions

VM conceived of the study, did the sequencing data analyses and interpretations and wrote the paper. DK and LP performed the cytogenetic analyses (GISH) and interpreted the data. All authors read and approved the final manuscript.

## Supplementary Material

Additional file 1 **Alignment of chloroplast *trn*L-F sequences**. A FASTA file comprising chloroplast *trn*L-F sequences is given. Click here for file

Additional file 2 **Alignment of nuclear GBSSI sequences**. A FASTA file comprising partial nuclear GBSSI sequences is given. Click here for file

Additional file 3 **Summary statistics for Ka/Ks analysis**. The file contains summary statistics for Ka/Ks ratios of coding portions of *Thinopyrum intermedium *GBSSI sequences. a) Ka/Ks values for each node in the tree are tabulated, b) Ka/Ks annotated evolutionary tree is given. Click here for file
