# Supplementary material for: On the genome constitution and evolution of intermediate wheatgrass (Thinopyrum intermedium: Poaceae, Triticeae)
Source: BMC Evol Biol. 2011 May 18;11:127. doi: 10.1186/1471-2148-11-127 (PMC3123223; doi:10.1186/1471-2148-11-127)

Additional file 3. Summary statistics for Ka/Ks analysis. a) Ka/Ks values for each node in the tree. b) Ka/Ks annotated evolutionary tree.

a)

Node #	Ka/Ks Branch 1	Ka Branch 1	Ks Branch 1	Ka/Ks Branch 2	Ka Branch 2	Ks Branch 2
1	7.3021	0.0073	$1e^{-10}$	0	0	$1e^{-10}$
2	0.0902	0.0040	0.0454	0	0	0.0146
3	0.0710	0.0032	0.0452	0	0	$1e^{-10}$
4	3.2097	0.0032	$1e^{-10}$	0.0911	0.0041	0.0451
5	0	0	$1e^{-10}$	4.0984	0.0041	$1e^{-10}$
6	2.0284	0.0020	$1e^{-10}$	0.1750	0.0052	0.0300
7	0.6570	0.0098	0.0149	0.1791	0.0107	0.0595
8	0	0	0.0221	0.2153	0.0016	0.0074
9	0.1669	0.0095	0.0570	0.5808	0.0101	0.0174
10	0	0	0.0377	0.3863	0.0139	0.0360
11	0.3008	0.0114	0.0380	0	0	0.0493
12	0.3065	0.0057	0.0186	0	0	0.0147
13	0.2763	0.0020	0.0074	0.2275	0.0021	0.0090

b)

Ka/Ks annotated evolutionary tree

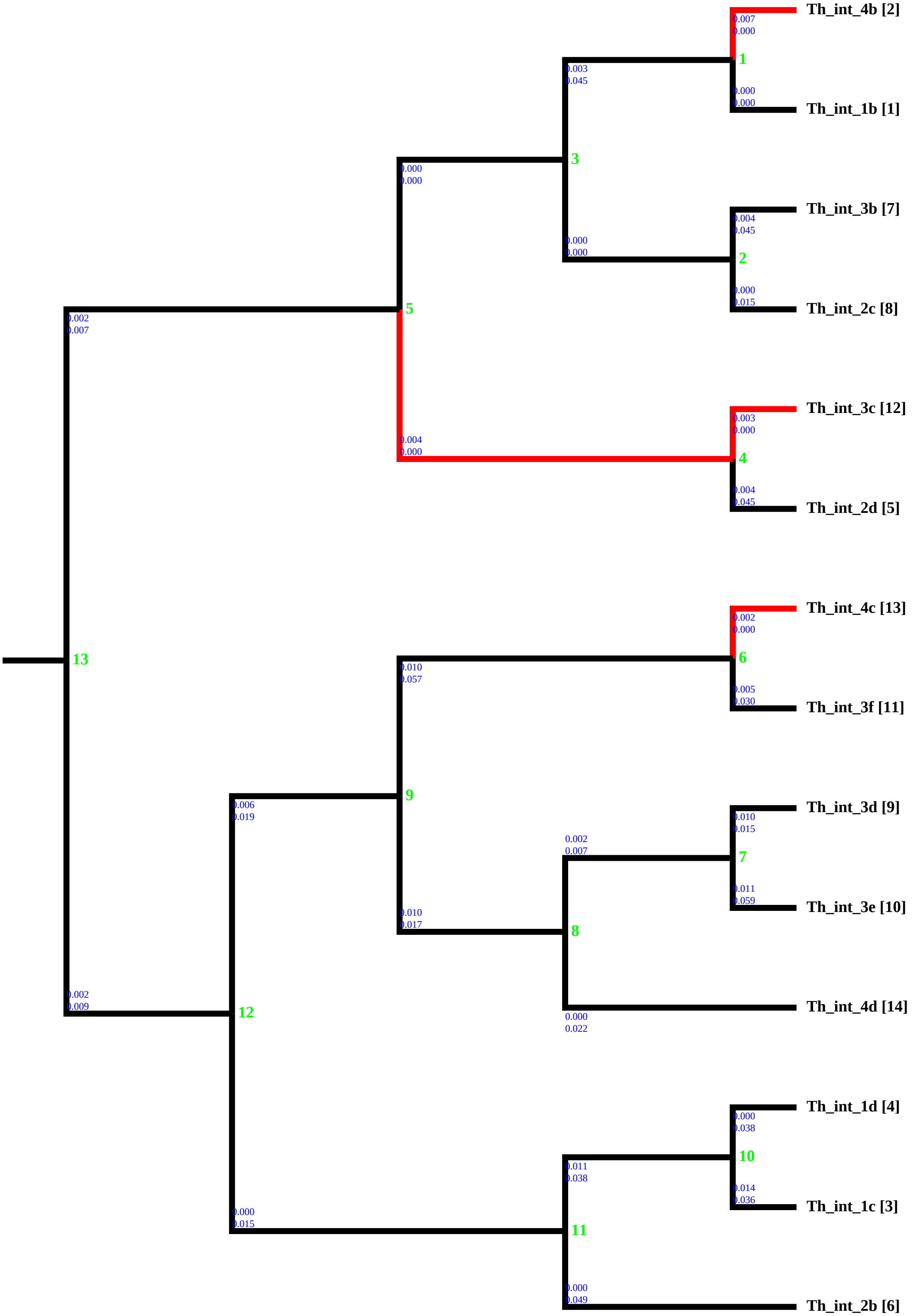

Supplement: Additional file 3 — Summary statistics for Ka/Ks analysis. The file contains summary statistics for Ka/Ks ratios of coding portions of Thinopyrum intermedium GBSSI sequences. a) Ka/Ks values for each node in the tree are tabulated, b) Ka/Ks annotated evolutionary tree is given. [file 1471-2148-11-127-S3.PDF]
